# Photonic synaptic transistors with new electron trapping layer for high performance and ultra-low power consumption

**DOI:** 10.1038/s41598-023-39646-w

**Published:** 2023-08-03

**Authors:** Taewoo Kim, Kwang-Seok Yun

**Affiliations:** Department of Electronic Engineering, Sogang University, 35 Baekbeom-ro, Mapo-gu, Seoul, 04107 Korea

**Keywords:** Engineering, Electrical and electronic engineering

## Abstract

Photonic synaptic transistors are being investigated for their potential applications in neuromorphic computing and artificial vision systems. Recently, a method for establishing a synaptic effect by preventing the recombination of electron–hole pairs by forming an energy barrier with a double-layer consisting of a channel and a light absorption layer has shown effective results. We report a triple-layer device created by coating a novel electron-trapping layer between the light-absorption layer and the gate-insulating layer. Compared to the conventional double-layer photonic synaptic structure, our triple-layer device significantly reduces the recombination rate, resulting in improved performance in terms of the output photocurrent and memory characteristics. Furthermore, our photonic synaptic transistor possesses excellent synaptic properties, such as paired-pulse facilitation (PPF), short-term potentiation (STP), and long-term potentiation (LTP), and demonstrates a good response to a low operating voltage of − 0.1 mV. The low power consumption experiment shows a very low energy consumption of 0.01375 fJ per spike. These findings suggest a way to improve the performance of future neuromorphic devices and artificial vision systems.

## Introduction

The traditional von Neumann method is unsuitable for processing large amounts of instantly generated and randomly moving information because it performs serial and sequential calculations through a single channel. In this traditional method, processing a large amount of data can lead to delays and failures, called von Neumann bottlenecks, and considerable energy consumption^1,2^. Therefore, researchers have focused on the human brain, which is highly integrated and can quickly and efficiently process information. A single synaptic event in the human brain consumes very low energy of approximately 10 fJ^3^. Brain-mimicking and neuromorphic synaptic devices have been of recent interest, and various low-power methods using the plasticity property have been published^4,5^.

Organic synaptic devices are attractive because of their light weight, large-area processing, and easy and low-cost fabrication^6,7^. In general, for devices using voltage-driven organic Field-Effect Transistor (FET) where the gate is modulated by an electric potential, synaptic properties have been implemented using ions moving slowly inside the electrolyte. Various high-functional devices or systems have been implemented using these properties^4,8,9^. An artificial tactile system with a skin sensor was developed using a ferroelectric layer or an ion gel^10,11^. Kim et al. reported an artificial afferent nerve by combining a pressure sensor and an ion gel^7^.

Recently, photonic synaptic devices have been reported, in addition to transistors that implement synaptic properties using gate voltage^6,12–15^. Compared to the voltage-driving method, photonic synaptic devices can have a wide bandwidth, fast transmission speed, and low power consumption^16^. These photonic synapse elements can also embody artificial vision. When the human eye receives visual information, the photoreceptors in the retina convert light into electric impulses, which are transmitted through nerves to the brain region that creates and stores images. In an artificial vision system, the device detects light and converts it into an electrical signal to generate and store light information^17^. The photonic synaptic transistor quickly converts an optical signal into an electrical signal and simultaneously exhibits excellent properties for information storage; therefore, it has recently been attracting attention as a device for constructing an artificial vision system^18^.

Many materials, such as organic semiconductors^18^, perovskites^19^, and environmentally-friendly biomaterials^20^, are used as light absorption layers. Among these, we used an inorganic halide perovskite (CsPbBr_3_), a semiconductor material with a hexagonal structure^12^. Because perovskites have high photoelectric efficiencies, they attract attention for optical devices such as solar cells^21^ and photodetectors^22^. However, they are very vulnerable to moisture, and their photoelectric efficiency decreases significantly when exposed to the atmosphere for a long time^23^. In this respect, CsPbBr_3_ made of inorganic materials has better stability than other organic–inorganic perovskites^24^.

Most light-driven organic synaptic devices consist of a double layer composed of a channel and a light absorption layer. When the light-absorption layer absorbs photons and generates an electron–hole pair, each channel and light-absorption layer is dominated by holes and electrons, respectively. The energy barrier caused by the different energy levels of each layer prevents the recombination of electrons and holes, resulting in synaptic phenomena where the drain current flows after light illumination is turned off^12,14^. However, most double-layer devices perform poorly in terms of the output current and “energy consumption per spike,” which are important parameters for measuring the performance of synaptic devices.

In this work, we dramatically improved the photoreactivity and energy consumption characteristics compared to those of the double-layered device by implementing a triple-layer device in which a new electron trapping layer (NETL) is adopted. The triple-layer device with a NETL lowers the electron–hole recombination rate compared to a double-layer device. This improvement enhances the carrier lifetime, which, in turn, contributes to an increase in channel conductivity.

We used 6,13-Bis(triisopropylsilylethynyl)pentacene (TIPS-pentacene, or simply TIPS), a p-type organic semiconductor material, as the channel layer and CsPbBr_3_ as the light absorption layer^12^. SnO_2_ was utilized as a NETL because it is simple to fabricate, has a valence band level that can create an energy barrier to trap electrons in conjunction with CsPbBr_3_, and provides a smooth thin-film surface^25,26^.

This paper presents a method capable of detecting very weak light. The approach boasts a higher photosensitivity than those previously reported for various photonic synaptic perovskite systems.

## Experimental section

### Material and fabrication methods

#### Material

0.3 M CsPbBr_3_ was prepared by dissolving PbBr_2_ (> = 98%, Sigma-Aldrich) and CsBr (99.999%, Sigma-Aldrich) in dimethyl sulfoxide (DMSO, 99.8%, SAMCHUN). TIPS was prepared by dissolving 10 mg 6,13-Bis(triisopropylsilylethynyl)pentacene (> 99%, TCI) and 10 mg polystyrene (Sigma-Aldrich) in 1 mL monochlorobenzene (> 99.7%, DAEJUNG). The SnO_2_ was prepared by diluting SnO_2_ colloidal solution (15% in H_2_O colloidal dispersion, Thermo Scientific) with deionized water to a final concentration of 5%.

#### Device fabrication

**Figure 1:**
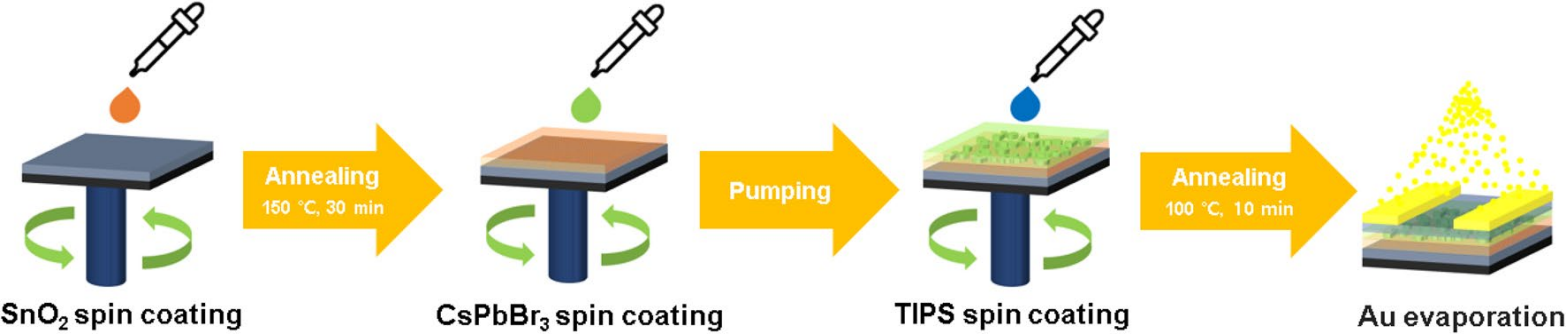
Schematic representation of the fabrication process of the triple-layer photonic synaptic transistor. The process begins with SnO_2_ spin coating, followed by consecutive spin coating of CsPbBr_3_ and TIPS, and finally Au electrode patterning.

Heavily doped n-type silicon with 100 nm SiO_2_ was used as the substrate. To prepare a clean substrate, ultrasonication was performed on the substrate for 10 min in the order of acetone, isopropyl alcohol, and deionized water. O_2_ plasma was applied to the substrate for 5 min. Next, the prepared SnO_2_ colloidal solution was dropped onto the substrate and spin-coated at 3000 rpm for 30 s, followed by annealing at 150 °C for 30 min. Next, the CsPbBr_3_ solution was spin-coated onto SnO_2_ at 4000 rpm for 60 s. The sample was placed in a vacuum pump to remove the solvent^12^. Then, the TIPS solution was spin-coated onto the perovskite layer at 1000 rpm for 10 s and annealed at 100 °C for 10 min. Finally, 50 nm of Au was thermally evaporated to form source and drain electrodes with a channel width of 1000 μm and a length of 50 μm. A simplified pictorial illustration of this fabrication process is shown in Fig. 1.

### Characterization and measurements

Electrical measurements were conducted in an ambient atmosphere using a Keithley 4200-SCS semiconductor parameter analyzer to gauge the synaptic performance of the device. For the light-dependent experiments, measurements were performed under dark conditions to avoid ambient light interference. UV–vis absorption measurements were performed using a JASCO V-750 spectrophotometer. The optical microscopy image of the device was observed using an Olympus BX51 microscope, and the nano images were observed using a JSM-7100F FE-SEM.

## Result and discussion

### Device structure and working principles of photonic synaptic transistors

**Figure 2:**
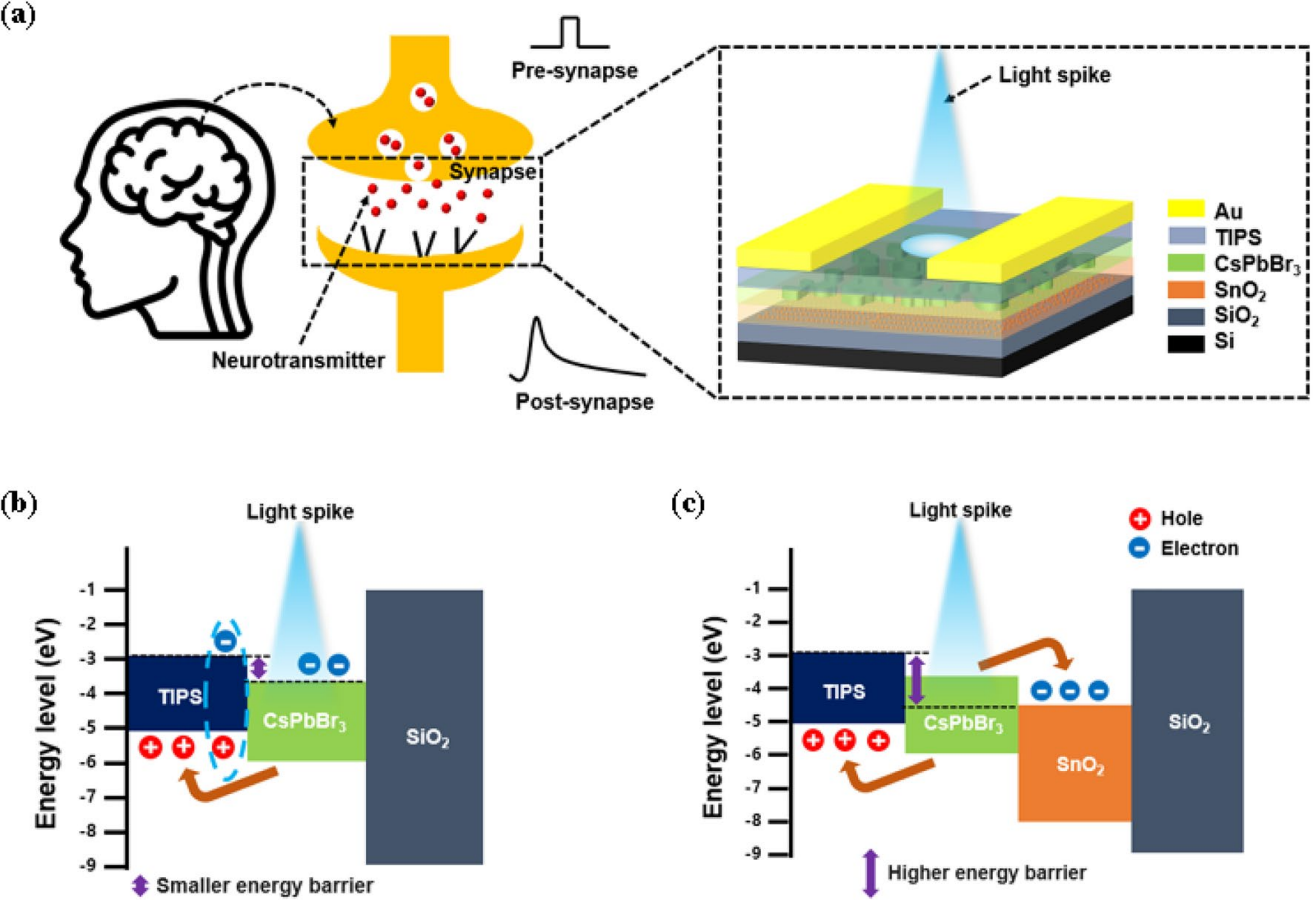
(**a**) Schematic image of biological synapse and our photonic synaptic transistor. (**b**) Energy band diagram of CsPbBr_3_/TIPS double-layer device. (**c**) Energy band diagram of SnO_2_/CsPbBr_3_/TIPS triple-layer device.

Understanding the biological synaptic mechanisms, illustrated on the left side of Fig. 2a, is helpful in developing a neuromorphic system. Synapses are links between neurons that exchange information from the presynaptic to the postsynaptic neuron^13,27^. In response to external stimuli, neurotransmitters bind to the receptors of postsynaptic neurons by creating postsynaptic currents. The right side of Fig. 2a shows the proposed photonic synaptic device that mimics this biological process. The external input is defined as a light spike, and the increased drain current is defined as an excitatory postsynaptic current (EPSC), which is crucial for the acquisition, transmission, and storage of data in synaptic devices^28^.

Energy band diagrams are shown in Fig. 2b and c to describe how photonic synaptic devices function in double and triple layers, respectively, where stairwise energy barriers are formed between the layers. Electron–hole pairs are generated in the CsPbBr_3_ perovskite when light illuminates the photonic synaptic transistor. In the double-layer device shown in Fig. 2b, the holes pass to the TIPS layer owing to the built-in potential bias^12^. However, electrons cannot cross easily owing to the barrier generated by the conduction band (CB) of CsPbBr_3_ with the lowest unoccupied molecular orbital (LUMO) level of the TIPS. Therefore, even if the light is off, because electrons are trapped in CsPbBr_3_, recombination with holes is prevented, and the current continues to flow. However, in the double-layer structure, the barrier is not large enough to prevent recombination for a long time, so the electrons trapped in CsPbBr_3_ recombine with the holes quickly, and no channel current flows. Differing from the double-layer device, in the SnO_2_/CsPbBr_3_/TIPS triple-layer device, the photogenerated electrons are directed to the SnO_2_ layer rather than the CsPbBr_3_ layer. Meanwhile, the holes migrate to the TIPS, similar to the double-layer structure. In this process, the physical distance and energy barrier between electrons and holes heighten compared to the double-layer device. This increase in distance and energy barrier is analogous to the use of electron and hole transport layers in solar cell research^29^. Therefore, owing to a diminished recombination rate in the triple-layer structure compared to the double-layer one, the number of holes contributing to the channel and the carrier lifetime increases. Using this energy-band engineering, the triple-layer structures exhibit improved optical responsiveness.

**Figure 3:**
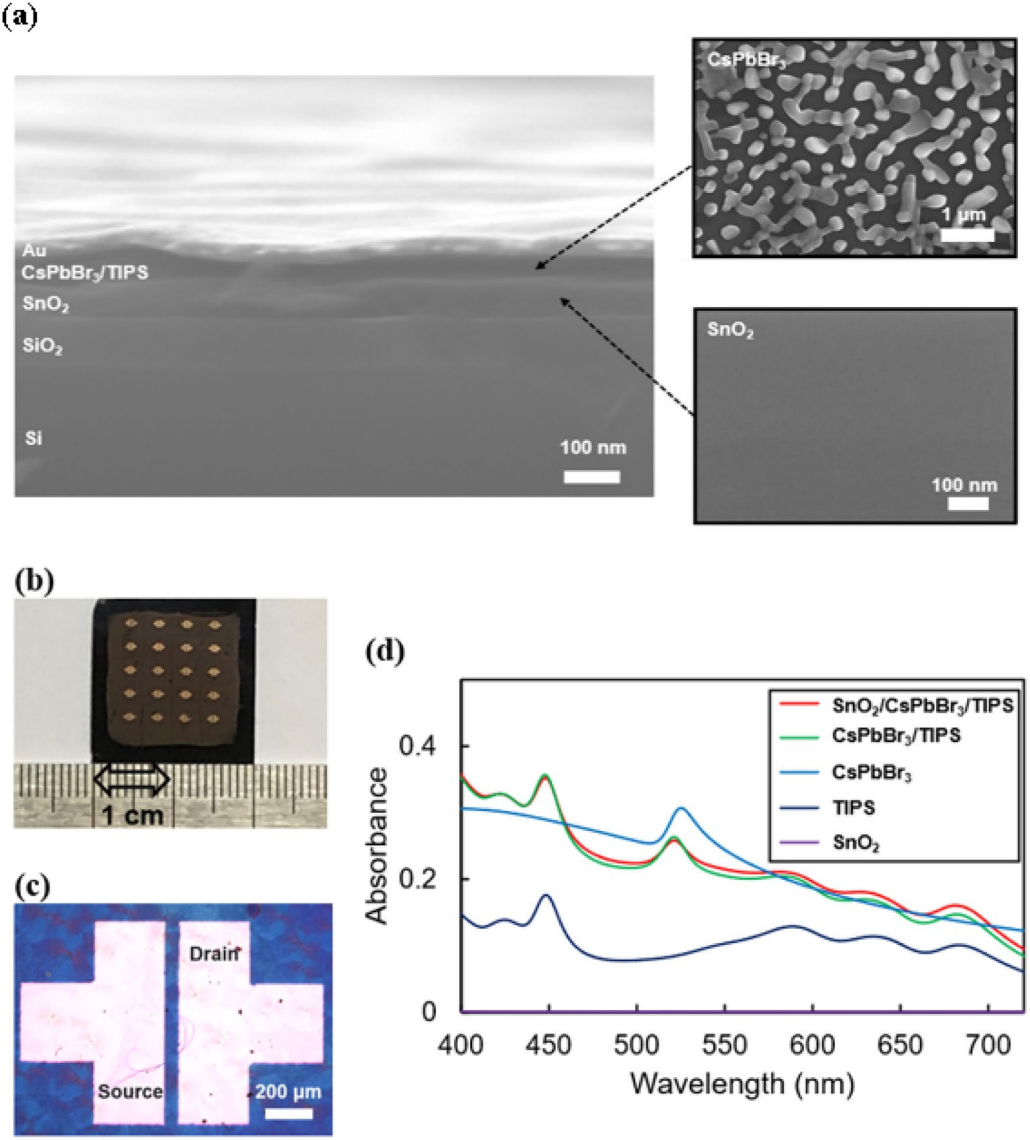
(**a**) Field emission scanning electron microscopy (FE-SEM) cross-sectional image of triple-layer device and surface image of SnO_2_ and CsPbBr_3_. (**b**) Photograph of 5 × 4 array of triple-layer device. (**c**) Optical microscopy image of source/drain electrode. (**d**) UV–vis absorption spectra of SnO_2_, TIPS, CsPbBr_3_, CsPbBr_3_/TIPS, SnO_2_/CsPbBr_3_/TIPS films, respectively.

A cross-section of the fabricated triple-layer synaptic transistor is shown in Fig. 3a. The device was fabricated using a solution process, except for the gold electrode. First, SnO_2_ was spin-coated onto the Si/SiO_2_ substrate. Subsequently, a CsPbBr_3_ solution, prepared by mixing CsBr and PbBr_2_ at a 1:1 molar ratio, was spin-coated on top of SnO_2_, yielding a CsPbBr_3_ layer with a thickness of approximately 60 nm. The thickness was judiciously chosen to achieve an optimal balance between light absorption, charge carrier transport, and compatibility with device scaling. After the CsPbBr_3_ layer deposition, TIPS was spin coated and thermal deposition of source/drain electrodes followed. As shown on the right side of Fig. 3a, SnO_2_-coated SiO_2_ shows a very homogeneous morphology^25,26^. In contrast, CsPbBr_3_ perovskite crystals manifest dispersed island-shaped grains. A structure with fewer grain boundaries is advantageous for preventing ion migration, causing a gate-field screening effect in the perovskite^30^ as ion immigration occurs at the grain boundary rather than at the grain. Furthermore, perovskite crystals react sensitively to weak light^12^. Further details about the fabrication process are provided in the Experimental section.

Figure 3b and c show an image of the fabricated synaptic transistor. The channel length is 50 μm, and the width is 1000 μm. Figure 3d shows the UV–vis spectra of SnO_2_, TIPS, CsPbBr_3_, CsPbBr_3_/TIPS, and SnO_2_/CsPbBr_3_/TIPS films. The absorption in SnO_2_ is extremely low compared to the other films; therefore, even if SnO_2_ is added to the CsPbBr_3_/TIPS film, there is no significant change in the peak value and light absorption^16^. The peak values of the CsPbBr_3_/TIPS and SnO_2_/CsPbBr_3_/TIPS films are similar to those of the TIPS film at 488 nm, indicating that TIPS determines the peak values. Because a high photocurrent gain can be obtained using the light of the wavelength closest to the peak^20^, for the light illumination experiment, we fixed the wavelength to 450 nm using an LED to give impulsive spikes on the device.

### Synaptic characteristics and performances of photonic synaptic transistors

**Figure 4:**
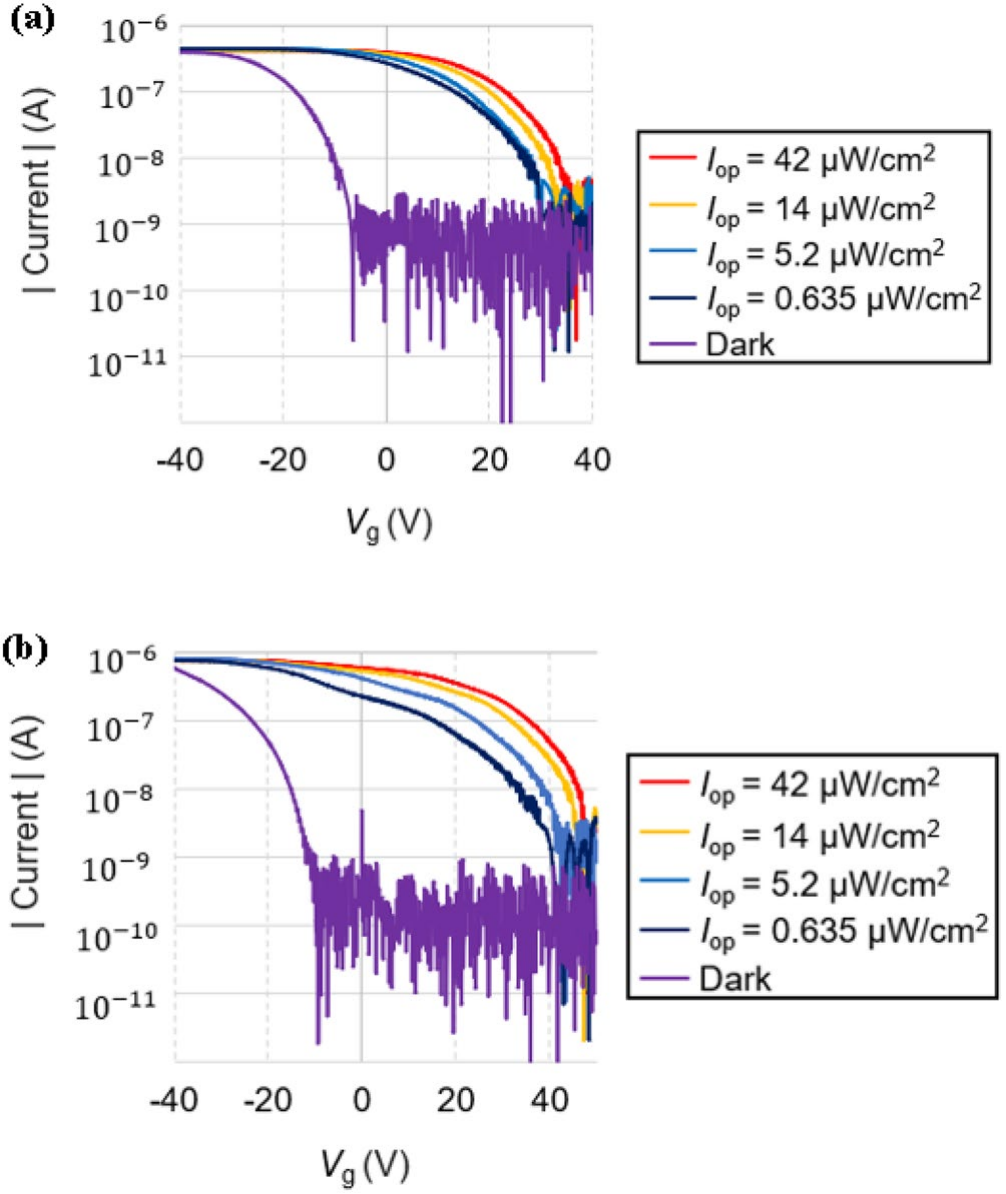
(**a**) Transfer curves under dark and various illumination conditions for double-layer and (**b**) triple-layer devices.

Figure 4a and b show the transfer curves of CsPbBr_3_/TIPS and SnO_2_/CsPbBr_3_/TIPS photonic synaptic transistors, respectively, measured at a drain voltage (*V*_d_) of − 40 V. When light is applied to each photonic synaptic transistor, the curve moves in the positive direction owing to the influence of the photo-generated electron–hole pairs. The curve moves to the right as it receives more intense light^12^, showing that the stronger the light, the higher the current obtained at the same gate voltage. When measuring the memory window, which represents the change in threshold voltage upon light illumination compared to the dark condition, the double-layer device at a defined light intensity (*I*_op_) of 0.635 μW/cm^2^ demonstrated a value of 38.9 V. This value is relatively high, particularly when considered in the context of the comparatively weak light intensity of 0.635 μW/cm^2^ employed in the experiments. However, the triple-layer device showed a much-improved memory window of 51.3 V. This is because, as described above, as the SnO_2_ layer was added, the number of surviving holes increased owing to the additional barrier to recombination.

**Figure 5:**
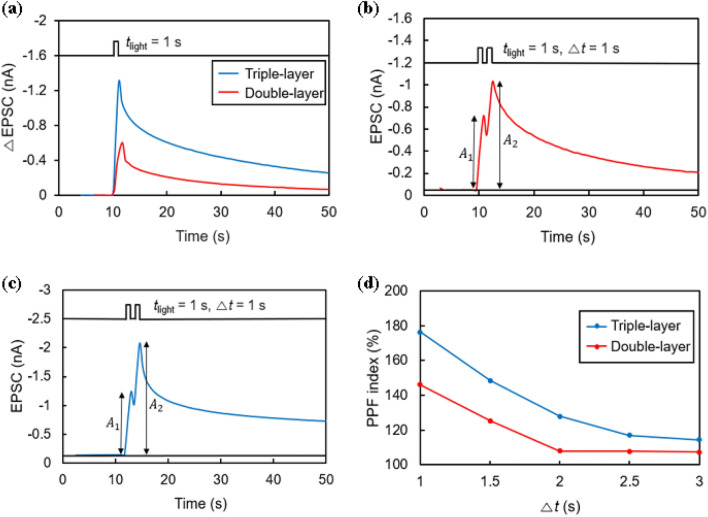
(**a**) Depiction of synaptic characteristics for both double-layer and triple-layer devices. (**b**) Reaction of EPSC to two successive light spikes in the double-layer device. (**c**) Reaction of EPSC to two successive light spikes in the triple-layer device. (**d**) Representation of PPF index as a function of spike interval (Δt) for both double-layer and triple-layer devices.

The synaptic characteristics of photonic synaptic transistors can be evaluated using the transient response of the current to light illumination^16^. Figure 5a shows the response currents in the double- and triple-layer devices. The time (*t*_light_) and intensity of the illuminated light were 1 s and 84 μW/cm^2^, respectively, at *V*_d_ = − 1 V. The gate voltage (*V*_g_) was fixed at 0 V. The current increment (ΔEPSC) is depicted in the graph instead of the raw current value because the ground levels of the two transistors are different: 0.035 nA for the double-layer and 0.547 nA for the triple-layer. EPSC peaks appear in both the double-layer and triple-layer transistors during the period in which light is applied, but ΔEPSC is 1.309 nA in the triple-layer device, which is 2.16 times larger than 0.605 nA in the double-layer device. These results show that the increased energy barrier in the triple-layer device effectively inhibits the recombination of electrons and holes.

When a light spike is continuously applied to the photonic synaptic transistor, the photocurrent generated by the second spike (*A*_2_ in Fig. 5b and c) exceeds that generated by the first spike (*A*_1_ in Fig. 5b and c). This phenomenon is known as paired-pulse facilitation (PPF)^13,31^ and is defined by Eq. (1):

1 $${\text{PPF }} = \, (A_{{2}} /A_{{1}} ) \times {1}00 \, \%$$

This phenomenon results from synaptic plasticity, which further increases the current if the second spike comes before the electrons and holes generated by the first spike are completely recombined. The PPF experiment was conducted at *t*_light_ = 1 s and *I*_op_ = 84 μW/cm^2^ intensity, and the time interval (Δ*t*) between the two spikes was varied from 1 to 3 s. Figure 5b and c show the EPSC graphs measured at Δ*t* = 1 s, showing higher EPSCs in the triple-layer device (Fig. 5c) than in the double-layer device (Fig. 5b). Figure 5d shows the PPF index measured at various Δ*t* values for the double- and triple-layer devices. In both cases, the PPF ratio decreases rapidly as Δ*t* increases, then gradually converges. For the double-layer device, the PPF decays from 146.06 to 107.25%, whereas the triple-layer device decays from 176.35 to 114.39%. The triple-layer device shows a higher PPF index at Δ*t* than the double-layer device. Owing to the larger energy barrier of the triple-layer device, the hole remains in the TIPS for a longer time before recombination with the electron, and thus a greater number of carriers remain in the channel until the second light arrives.

**Figure 6:**
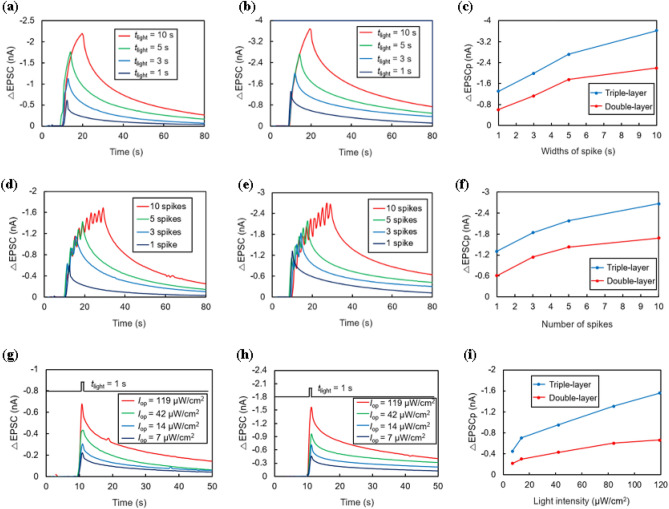
(**a**–**b**) ΔEPSC as a function of spike widths for double-layer and triple-layer devices, respectively. (**c**) Peak of ΔEPSC (ΔEPSCp) as a function of spike widths. (**d**–**e**) ΔEPSC when influenced by varying numbers of consecutive light spikes in double-layer and triple-layer devices, respectively. (**f**) ΔEPSCp as a function of spike number for double-layer and triple-layer devices. (**g**–**h**) ΔEPSC measurements at various light intensities for double-layer and triple-layer devices, respectively. (**i**) ΔEPSCp as a function of light intensity for double-layer and triple-layer devices.

Analogous to how repeated information can be better memorized in the human brain, the transition from short-term potentiation (STP) to long-term potentiation (LTP) when a longer and stronger stimulus is applied is also an important characteristic of synaptic devices^10^. In Fig. 6a and b, changes in the EPSC can be observed according to the spike widths of light. The light intensity was 84 μW/cm^2^ in this experiment. Carriers are continuously created with continuous light input, resulting in a higher current. In Fig. 6d and e, the change in EPSC is observed while changing the number of consecutive light spikes from 1 to 10 under the conditions of *t*_light_ = 1 s, Δ*t* = 1 s, and *I*_op_ = 84 μW/cm^2^. Similar to the previous experiment, the higher the number of spikes, the higher the peak current. Finally, Fig. 6g and h show the results of experiments applied to double-and triple-layer photosynaptic transistors for 1 s while varying the light intensity. A higher EPSC can be obtained because more electron–hole pairs are generated as the light intensity increases. In addition, owing to the increased energy barrier, experiments using triple-layer devices in Fig. 6b, e, and h show higher photoreactivity than results from double-layer devices in Fig. 6a, d, and g. The trend of change in the peak value of each experiment can be compared in Fig. 6c, f and i, where it can be seen that the slope of the curve becomes flatter as the stimulus (spike widths, number of light spikes, and light intensity) applied to the device increases. This is because the strong stimulus causes the electrons in CsPbBr_3_ in the photon absorption layer to reach a high density, and the new photogenerated holes directly recombine with the electrons before moving into the TIPS layer^19^. From the results shown in Fig. 6i, it is evident that the triple-layer device exhibits a response rate that is more than twice as high as that under both low and high light intensities. This increased response is predominantly attributed to the energy band mechanism intrinsic to the triple-layer structure, which effectively lowers the recombination rate of electrons and holes. As a result, this implies a significantly larger number of residual channel carriers, or holes, available to contribute to the channel current.

One peculiar result of these experiments is that the triple-layer device shows a relatively steady increase in EPSC with a lower tendency toward saturation than the double-layer device. This is because, as electrons generated in the triple-layer device move to the SnO_2_ layer, the recombination of electrons and holes in the CsPbBr_3_ layer is reduced, and the lifetime of the holes is increased. Owing to this increased hole lifetime, the maximum photocurrent that can be reached is higher than that of the double-layer device.

**Figure 7:**
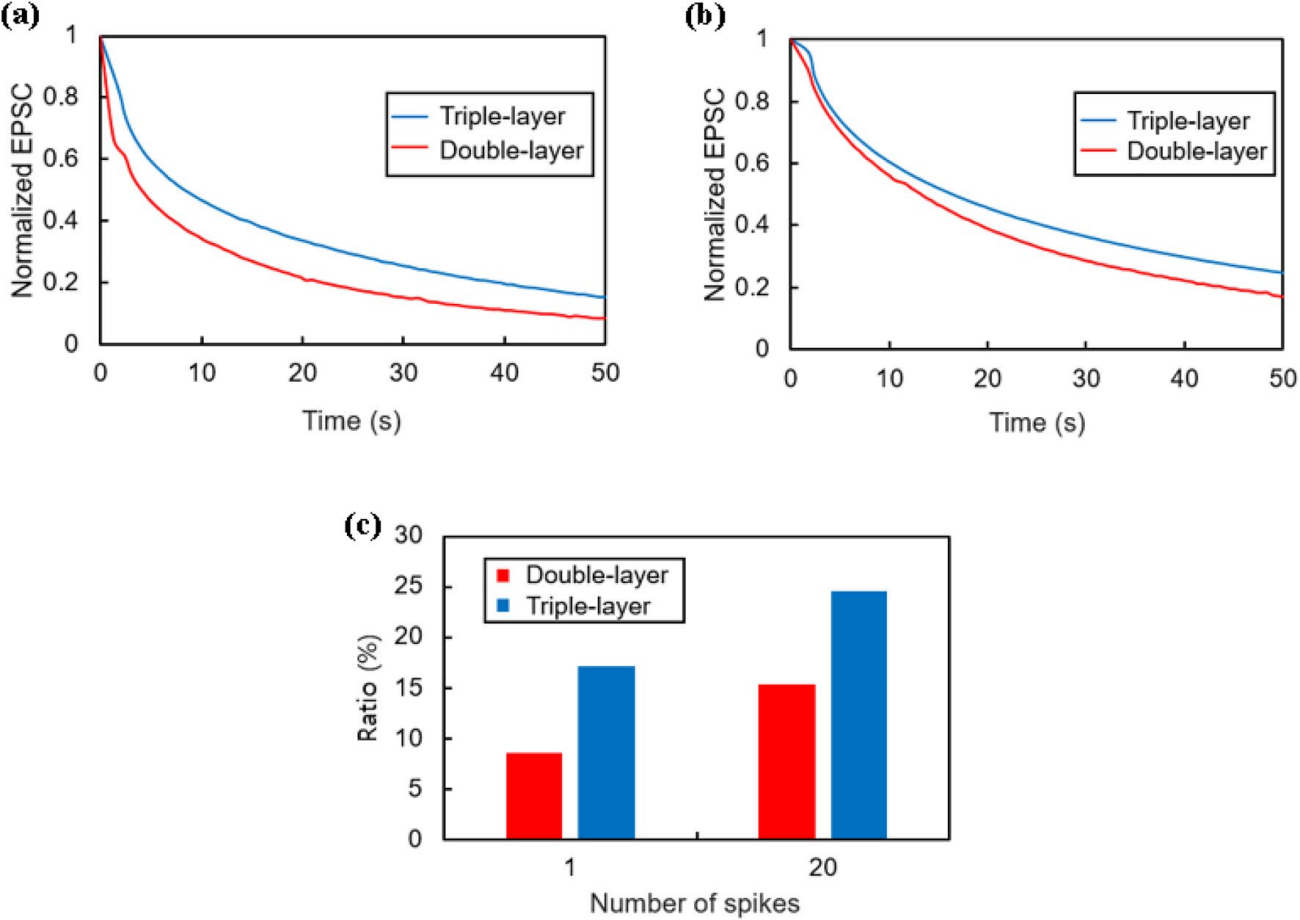
Depiction of EPSC decay characteristics and memory attributes for double-layer and triple-layer devices: (**a**) EPSC decay after the application of a single light spike; (**b**) EPSC decay following 20 consecutive light spikes; (**c**) Comparison of memory characteristics recorded at 50 s after the light is turned off for both device types.

In the transistor with the added NETL, both the increment in the EPSC and the effect of prolonging the retention time were observed. Figure 7a shows the decay of the EPSC in both double-layer and triple-layer devices under the STP condition (single spike). Because the peak EPSC values ​​of the two transistors are different, each peak value is normalized to 1. Fifty seconds after the light is turned off, the EPSC of the double-layer device retains 8.505% of the peak value, while 15.331% of the peak value is retained in the triple-layer device. This shows that the transistor with the SnO_2_ layer has better retention characteristics than the double-layer device under the same operating voltage and light conditions. The LTP results were obtained by applying 20 consecutive spikes (Fig. 7b). For the double-layer device, the EPSC decreases to 17.096% of the peak current value 50 s after the light is turned off, but in the case of the triple-layer device, it decreases only to 24.567%. This result also indicates that the triple-layer device has better retention characteristics than the double-layer device, and that the retention time is longer in the LTP condition than in the STP condition. The graph in Fig. 7c compares the memory characteristics for the double- and triple-layer devices at 50 s after the light is turned off. Overall, our device's relatively high EPSC and long retention time demonstrate higher performance under the same voltage and light conditions and also provide a significant advantage over the double-layer for long-term image memory in vision systems.

**Figure 8:**
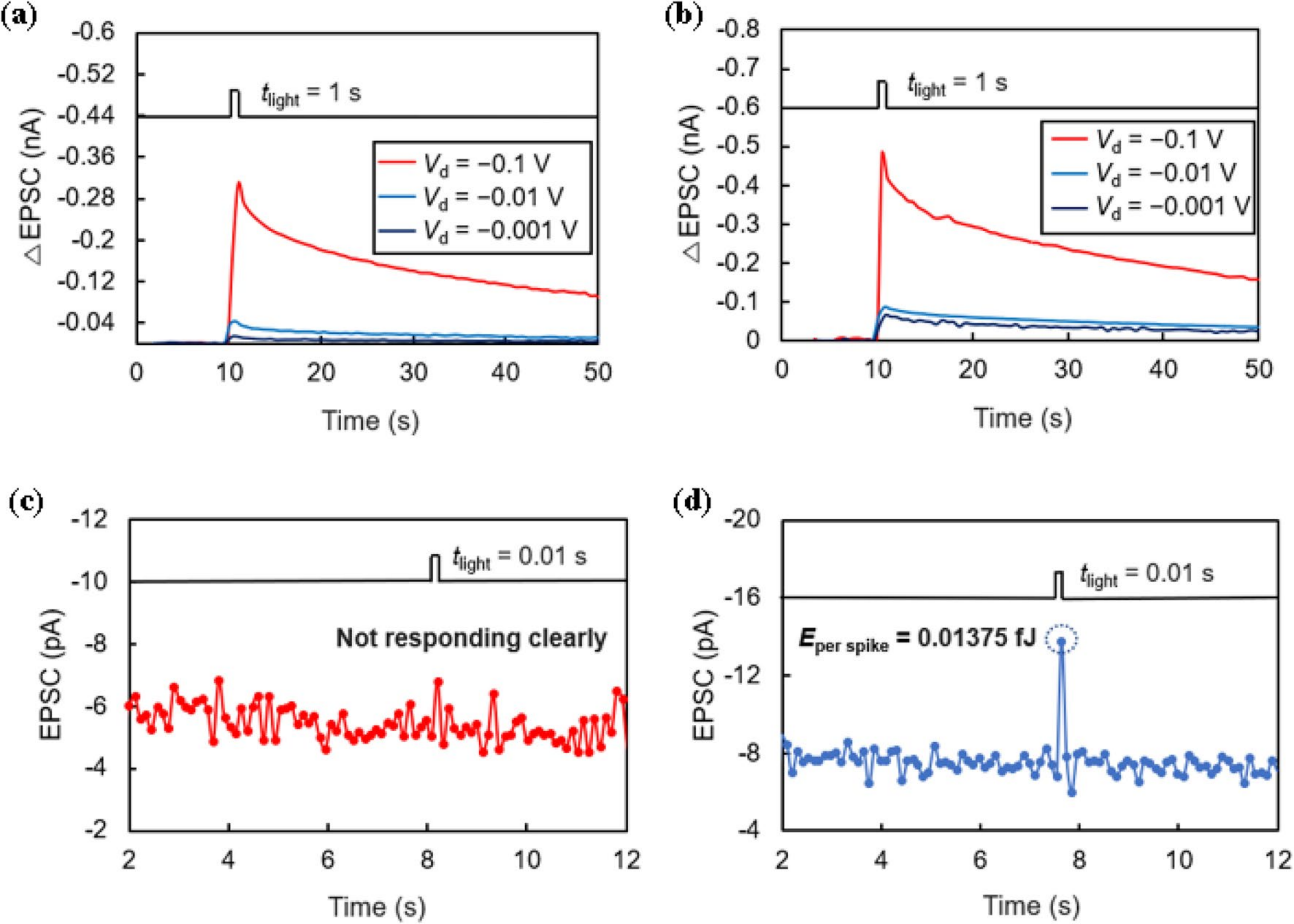
Variation in ΔEPSC under different operating conditions: (**a**) Double-layer device and (**b**) triple-layer device under varying operating voltages (*t*_light_ = 1 s, *I*_op_ = 42 μW/cm^2^). EPSC generated under extreme condition (*V*_d_ = − 0.1 mV, *t*_light_ = 0.01 s, *I*_op_ = 0.17 μW/cm^2^) for (**c**) double-layer and (**d**) triple-layer devices.

To investigate the performance under low power conditions^32^, the device operation was measured while lowering the operating voltage. Figure 8a and b present ΔEPSC obtained by lowering the operating voltage from − 0.1 to − 0.001 V under the condition of *t*_light_ = 1 s and *I*_op_ = 42 μW/cm^2^. Although the current rapidly decreases as the operating voltage decreases, both the double- and triple-layer devices respond to light spikes in this voltage range. To test the power consumption limit of our device, we prepared an extreme condition of very low operating voltage, low spike width, and low intensity of light (*V*_d_ = − 0.1 mV, *t*_light_ = 0.01 s, *I*_op_ = 0.17 μW/cm^2^). Under these conditions, the double-layer device cannot derive a distinguishable peak value, and only noise can be observed (Fig. 8c). Conversely, Fig. 8d shows that the triple-layer device undergoes a photoreaction, indicating that more holes survive in the channel in the triple-layer device than in the double-layer device. The energy consumption of this single spike is calculated using the following formula:^33–35^

2 $$E_{{\text{per spike}}} = V_{{\text{d}}} \cdot I_{{{\text{peak}}}} \cdot t_{{{\text{light}}}}$$

Our synaptic device has a very low energy consumption of 0.01375 fJ, which is about 1000 times lower than the approximately 10 fJ occurring in the human brain^13^. A comparison with recently published results is presented in Table 1.

**Table 1:** Comparison of power consumption of the photonic synaptic transistors recently reported.

Light absorption layer	Channel layer	Additional carrier trapping layer	Wavelength (nm)	Power consumption	Light intensity (μW/cm^2^)	Ref
CsPbBr_3_	PDPP4T	–	450	1.3 fJ	650	^13^
CsBi_10_I_10_	SWCNTs	–	450	~ 1 pJ	5	^16^
CsPbBr_3_ QDs	PDPP4T	–	450	0.5 fJ	50	^19^
Chlorophyll-a	SWCNTs	–	665	17.5 fJ	500	^20^
Natural carotene	PDPP4T	–	450	0.0034 fJ	–	^32^
CsPbBr_3_	C_8_–BTBT	–	365	0.114 fJ	0.5	^33^
Nitride acid-treated C_3_N_4_	Pentacene	–	365	18.06 fJ	380	^36^
CsPbBr_3_	TIPS-pentacene	SnO_2_	450	0.01375 fJ	0.17	This work

## Conclusions

In conclusion, we achieved a high-performance synaptic effect in a triple-layer device by integrating the widely studied CsPbBr_3_ and introducing the NETL material SnO_2_. Our innovative triple-layer configuration, different from the conventional double-layer where electrons were trapped in the perovskite, allowed the confinement of electrons in the NETL situated beneath the perovskite layer. This arrangement formed a higher energy barrier, reducing the electron–hole recombination rate and leading to a marked enhancement in the EPSC. The device demonstrated various synaptic effects such as PPF, STP, and LTP, and showed extremely low energy consumption in the power consumption test, marking its potential for applications demanding energy efficiency. The practicality of mass-production through straightforward and cost-effective solution processes like spin-coating further increases its value. While our device exhibited reasonable stability, showing photoresponsivity for about a week, longer-term stability was compromised. Future work will aim to improve this aspect, potentially through the implementation of capping materials or novel packaging technologies. Moving forward, we acknowledge the ongoing research towards the development of materials with superior photoreactivity and less toxicity for optimization of these devices. Our work contributes significantly to the construction of networks essential for future neuromorphic computing and artificial vision systems. It lays a promising foundation for potential applications including small-scale devices, mobile applications, and complex visual recognition tasks in areas like robotic systems, further substantiating the potential of our findings^36^.

## Data Availability

The datasets used and/or analyzed during the current study available from the corresponding author on reasonable request.
